# Application of near-infrared spectroscopy to assess the effect of the cupping size on the spatial hemodynamic response from the area inside and outside the cup of the biceps

**DOI:** 10.1371/journal.pone.0302828

**Published:** 2024-05-09

**Authors:** Pu-Chun Mo, Cheng-Feng Lin, Yameng Li, Manuel E. Hernandez, Jen-Chieh Liao, Isabella Yu-Ju Hung, Yih-Kuen Jan

**Affiliations:** 1 Department of Kinesiology and Community Health, University of Illinois at Urbana-Champaign, Urbana, Illinois, United States of America; 2 Department of Biomedical Engineering, National Cheng Kung University, Tainan, Taiwan; 3 Department of Physical Therapy, National Cheng Kung University, Tainan, Taiwan; 4 Department of Biomedical and Translational Science, University of Illinois at Urbana-Champaign, Urbana, Illinois, United States of America; 5 Department of Neurosurgery, Chi Mei Hospital Chiali, Tainan, Taiwan; 6 Department of Nursing, Chung Hwa University of Medical Technology, Tainan, Taiwan; Beijing University of Chinese Medicine, CHINA

## Abstract

Cupping therapy is a popular intervention for improving muscle recovery after exercise although clinical evidence is weak. Previous studies demonstrated that cupping therapy may improve microcirculation of the soft tissue to accelerate tissue healing. However, it is unclear whether the cupping size could affect the spatial hemodynamic response of the treated muscle. The objective of this study was to use 8-channel near-infrared spectroscopy to assess this clinical question by assessing the effect of 3 cupping sizes (35, 40, and 45 mm in inner diameter of the circular cup) under −300 mmHg for 5 min on the muscle hemodynamic response from the area inside and outside the cup, including oxyhemoglobin and deoxy-hemoglobin in 18 healthy adults. Two-way factorial design was used to assess the interaction between the cupping size (35, 40, and 45 mm) and the location (inside and outside the cup) and the main effects of the cupping size and the location. The two-way repeated measures ANOVA demonstrated an interaction between the cupping size and the location in deoxy-hemoglobin (*P* = 0.039) but no interaction in oxyhemoglobin (*P* = 0.100), and a main effect of the cup size (*P* = 0.001) and location (*P* = 0.023) factors in oxyhemoglobin. For the cupping size factor, the 45-mm cup resulted in a significant increase in oxyhemoglobin (5.738±0.760 μM) compared to the 40-mm (2.095±0.312 μM, *P*<0.001) and 35-mm (3.134±0.515 μM, *P*<0.01) cup. Our findings demonstrate that the cupping size and location factors affect the muscle hemodynamic response, and the use of multi-channel near-infrared spectroscopy may help understand benefits of cupping therapy on managing musculoskeletal impairment.

## Introduction

Cupping therapy is a popular intervention for improving muscle recovery and reducing muscle stiffness among healthy people and athletes [1–3]. To date, evidence for the effectiveness of cupping therapy on managing various musculoskeletal impairments remains weak [4,5]. Researchers indicate the need to improve the understanding of the underlying physiological mechanism of cupping therapy for improving clinical effectiveness [1,2,6].

Al-Bedah et al. proposed several potential mechanisms to explain benefits of cupping therapy [6]. Among these theories, the nitric oxide theory aligns well with current evidence on an increase in skin and muscle blood flow after cupping therapy [7–9]. The nitric oxide theory indicates that cupping therapy could stimulate a release of nitric oxide for increasing blood flow at the area affected by negative pressure of cupping therapy [6]. Increased blood flow of the treated area after cupping therapy may accelerate the removal of metabolic wastes and the supply of oxygen and nutrients, thereby improving musculoskeletal healing [6,10,11]. In order to induce an effective response from the microcirculation of the treated area, a proper combination of cupping pressure, duration and size is needed [12,13]. Several studies have attempted to demonstrate that the intensity of cupping therapy affects the microvascular responses and pointed out that an insufficient intensity of cupping therapy may not benefit treated area [1,2,7,8].

The cupping size (the cup size used in cupping therapy) is a more accessible factor for clinicians to easily manipulate for inducing needed microvascular response to meet the need of the patient [13,14]. The effects of different cupping sizes on the microvascular response and soft tissue mechanical property have been investigated in the literature [2,7,14,15]. He et al. compared skin blood flow response to three sizes of the circular cup (inner diameters at 35, 40, and 45 mm) and found that the 45-mm cup was more effective on increasing skin blood flow compared to the other two sizes [14]. The authors provide first evidence on the effect of cup size on modulating skin blood flow responses after cupping therapy. In order to shed light on the underlying physiological mechanism associated with increased blood flow, Hou et al. used wavelet analysis to characterize the changes in blood flow oscillations after cupping therapy. The authors found that an increase in skin blood flow is associated with an increase in power of the 0.0095–0.02Hz frequency band that is related to the metabolic activity [7]. The finding indicates that the metabolic related regulation could be modulated through cupping therapy. In addition to investigate microvascular responses after cupping therapy, Jan et al. performed the first study using elastographic ultrasound to explore the changes in muscle stiffness after cupping therapy. The results demonstrated that the 45-mm cup was more effective on reducing stiffness of the deep layer of the muscle compared to the 40-mm and 35-mm cup [2]. These studies have provided initial evidence that the application of different cupping sizes to the skin under the same negative pressure and duration would induce different responses from the treated area. To date, there is no study investigating the muscle blood flow response to different cupping sizes.

Tham et al. demonstrated that the area inside the cup is under high tensile stress and the area under the cup rim is under compressive stress using computational simulation (finite element method) to investigate the mechanical stress acting on the soft tissue during cupping therapy, [15]. From their results, both tensile and compressive forces are applied through cupping therapy to the treated area. The complex behavior of mechanical forces from cupping therapy may partly contribute to diverse responses from different studies tested in patients with different mechanical properties of treated soft tissues [4]. Research studies are needed to explore the spatial response (eg. areas inside and outside the cupping cup) of the treated soft tissue to cupping therapy with various sizes of the cup.

Near-infrared spectroscopy (NIRS) can measure tissue oxyhemoglobin and deoxy-hemoglobin, and can be used to study the spatial hemodynamic response of the muscle to cupping therapy [16,17]. NIRS has been used to measure the change in muscle hemodynamic responses after cupping therapy [18,19]. Kim et al. demonstrated that cupping therapy could increase deoxy-hemoglobin and oxyhemoglobin at the area inside the cup during cupping therapy [18]. Li and colleagues demonstrated that different combinations of cupping pressures and durations could significantly affect the hemodynamic response in the area inside the cupping cup rim [9]. To date, there is no study comparing the effect of cupping size on the muscle hemodynamic response using near-infrared spectroscopy.

Therefore, the objective of this study was to investigate the effect of cupping size on the spatial hemodynamic response of the muscle using multi-channel NIRS. The use of multi-channel NIRS would allow us to examine the hemodynamic response from both the areas inside and outside the cupping cup. We hypothesized that a larger size of the cup could induce a larger hemodynamic response of the muscle, including higher oxyhemoglobin and higher deoxy-hemoglobin concentrations. The second hypothesis was that a larger hemodynamic response from inside the cup would induce a larger hemodynamic response outside the cup. The findings from this study would provide initial evidence on the effect of cupping size on the muscle hemodynamic response as well as the association of the areas inside and outside the cup. To the best of our knowledge, this is the first study investigate the effect of the cupping size on the hemodynamic response of the muscle.

## Methods

A repeated measures study design was used to assess the effect of cupping sizes on the spatial hemodynamic response of the muscle. The repeated measures design was chosen to minimize the influence from the between subject characteristics in this study. The participant was blinded to the selection of the cup size to minimize the influence from other factors on the muscle hemodynamic response [4]. Data collected from this study were coded and were blinded to people who performed data analysis of NIRS signals. This study was part of a series of studies on assessing muscle hemodynamic responses under various cupping conditions. The data from this study have not been reported elsewhere.

### Participants

The inclusion criteria were between 18 to 40 years of age. The exclusion criteria were: 1) non-blanchable response of the red skin areas over the biceps and triceps (the dominant side), 2) open wounds, scars, or tattoos over the tested area, 3) diagnosed ischemic heart diseases (diagnosed coronary insufficiency, arrhythmia, and heart failure), 4) diagnosed diabetes, 5) any vascular diseases, 6) any neuromuscular impairments, 7) smokers, and 8) hypertension (systolic blood pressure >140mmHg or diastolic blood pressure >90mmHg). The distance from the acromion to the medial epicondyle of the humerus bone was used to define the arm length. The sample size estimation was based on previous studies [2,7,14] demonstrating significant differences in skin blood flow and tissue property after different cup sizes for choosing a large effect size at 0.4, power at 0.8, and alpha level at 0.05 for a sample size of 12 (G Power, ANOVA, repeated measures, within-between interaction). Because there are six possible sequences of 3 cup sizes (i.e., 35-40-45, 35-45-40, 40-45-35, 40-35-45, 45-35-40, and 45-40-35), the sample size needs to be a multiple of 6. In order to minimize the order and time effect, a sample size of 18 was chosen for this study. In this study, all subjects were recruited from the University of Illinois at Champaign-Urbana through flyers and word-of-mouth. All participants signed the informed consent approved by the University of Illinois at Urbana-Champaign Institutional Review Board on May 18, 2022 (#22900). The subject recruitment was performed between May 22, 2022 and August 10, 2022.

### Instrumentation

In this study, an automatic suction pump was used to create negative pressure inside the cupping cup (P1000-PCS, California Medical Device Manufacturing Facility, CA). This type of cupping therapy is usually called dry cupping. A pre-determined negative pressure value could be achieved by adjusting the setting of the cupping device (ranged from 0 to −760 mmHg). This study used three different cupping sizes, including 35 mm, 40 mm, and 45 mm (the inner diameter of the cup). The selection of these 3 sizes is because these sizes are commonly used on treating extremity muscles [2,7]. The total rim width is about 8 mm including the thickness for one side at 4mm and the other side at 4mm. For the 45-mm cup, the outer diameter is 53 mm. The negative pressure was selected at −300 mmHg for 5 minutes. This common setting of cupping therapy was demonstrated to be sufficient to induce the hemodynamic response of the skin and muscle [2,7,9].

**Fig 1:**
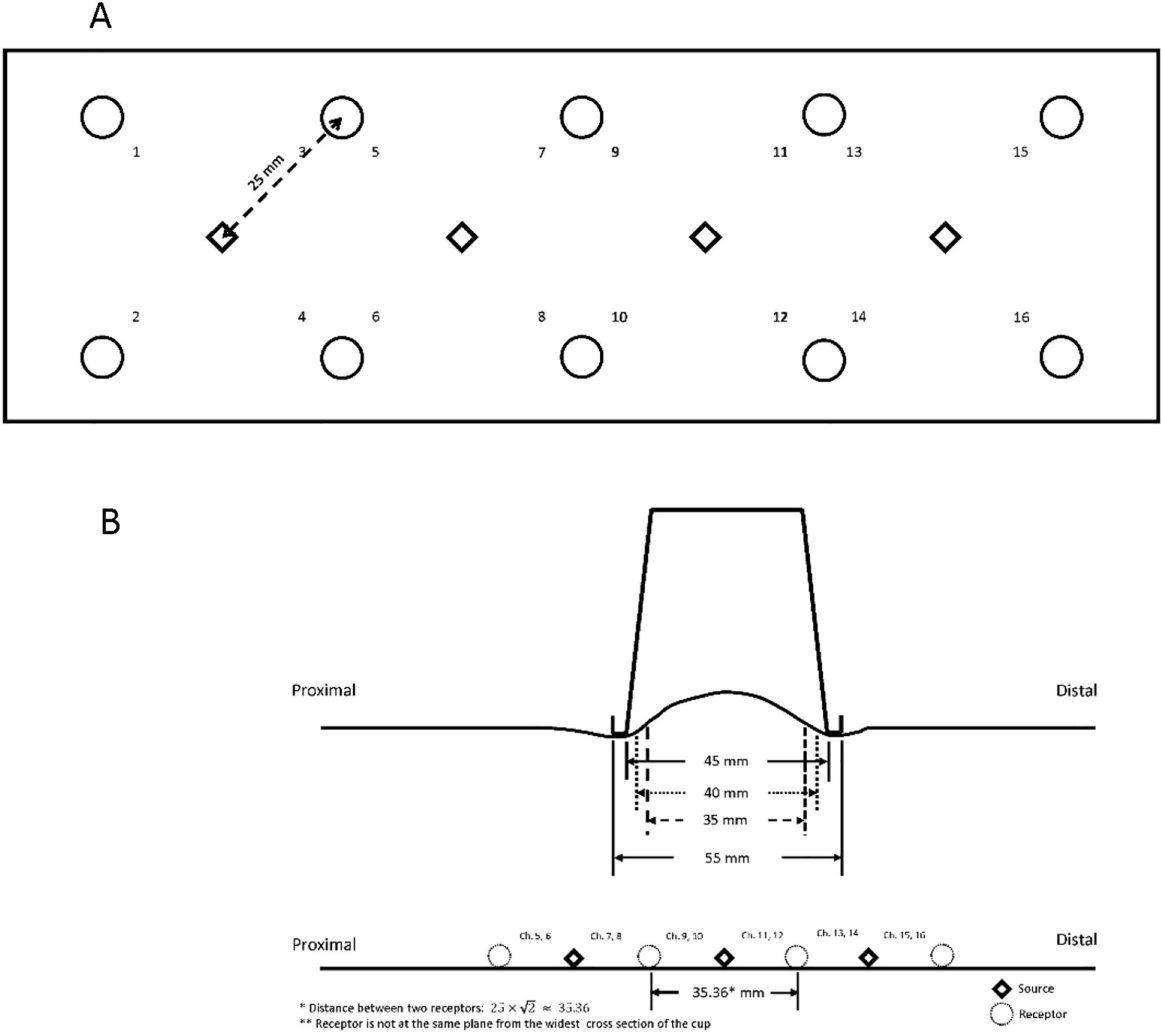
The near-infrared spectroscopy and the location of cupping therapy. A) The sensor pad of the near-infrared spectroscopy shows 4 emitters and 10 receivers for a total of 16 channel. Channels 9, 10, 11 and 12 are used for measuring the muscle hemodynamic response inside the cup and channels 7, 8, 13 and 14 are used to measure the muscle hemodynamic response outside the cup before and after cupping therapy. B) The cupping cup is placed on the biceps muscle under −300 mmHg for 5 minutes. Three sizes of circular cup (35, 40, and 45 mm in inner diameter) were tested.

A 16-channel NIRS system (fNIR Imager 1000, fNIR Devices LLC, Potomac, MD) was used to measure the muscle hemodynamic response, including the concentration of oxyhemoglobin and deoxy-hemoglobin [9,20]. The sensor band included ten photodetectors (receptors) and four LED lights (sources). Four receptors surround each source, and the distance between the receptor and its adjacent source is 25 mm (Fig 1A). Three cup sizes fall within the area covered by the channels of 9, 10, 11, and 12 (Fig 1B). The equal area outside the channels 9, 10, 11, and 12 are used to measure the hemodynamic response from outside the cup, that is, channels 7, 8, 13, and 14. Because the distance between the NIRS sensors were not adjusted to match each of 3 cup sizes, the bias may exist. However, this design (equal area for the inside and outside cup for all 3 cup sizes) may reduce the influence of spatial variation of microvascular system [21]. The source of the NIRS system was sampled at 2 Hz. Through the Modified Beer-Lambert Law, the relative concentration of oxyhemoglobin (Δ[HbO_2_]) and deoxy-hemoglobin (Δ[Hb]) can be calculated [22,23]. The detectable depth of the NIRS device is emanated at 12.5 mm [24,25]. The raw fNIRS signals were low-pass filtered within a finite impulse response filter of cut-off frequency at 0.14 Hz to eliminate possible respiration and heart rate signals and unwanted high frequency noise. The NIRS signal of 5-min pre-cupping period was used to calculate the relative change of NIRS signals after cupping therapy.

### Experimental procedures

All the protocols and examinations were conducted in Rehabilitation Engineering Laboratory at the University of Illinois at Urbana-Champaign. All participants were asked to lie supine on a mat table in a relaxing condition. Three cupping protocols with the cup size at 35 mm, 40 mm, and 45 mm (in inner diameter) were tested on the same day. A 40-minute washout period was provided between each protocol. The sequence of three protocols was determined randomly but with an equal number of subjects between six possible sequences (i.e., 35-40-45, 35-45-40, 40-45-35, 40-35-45, 45-35-40, and 45-40-35). The intervention took 5 minutes with −300 mmHg negative pressure. After removing the cupping cup, the post-cupping muscle hemodynamic response was measured using NIRS. At the end of each protocol, the subjective feeling was asked to understand the feeling of the participant. The participant was also instructed to call the lab if he/she had lasting pain after cupping therapy. It took three hours for a subject to complete all protocols.

**Fig 2:**
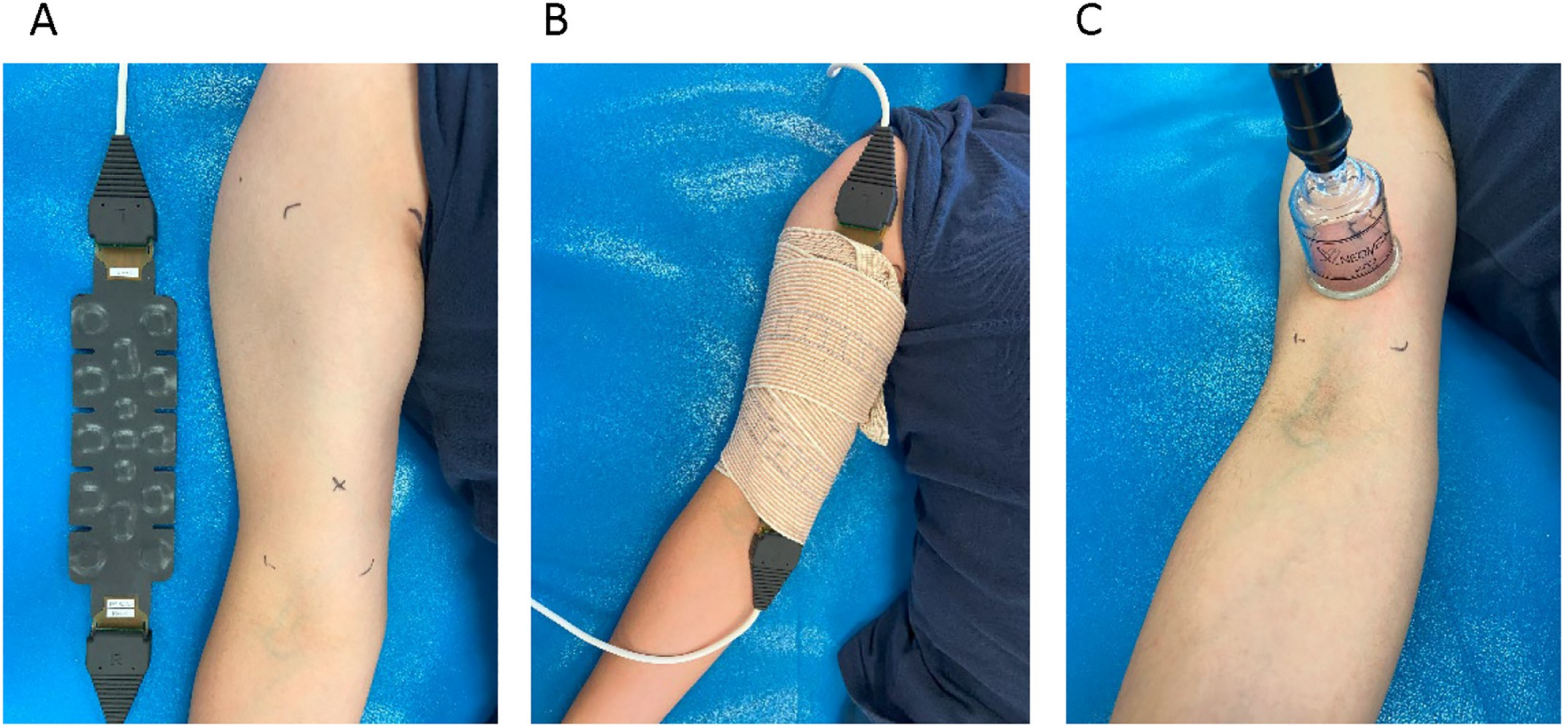
Photographs of the near-infrared spectroscopy sensor and cupping cup. (A) Near-infrared spectroscopy sensor. (B) The elastic band is used to wrap the sensor around the arm. (C) The cupping cup is used to apply negative pressure on the biceps muscle under −300 mmHg for 5 minutes.

Before the cupping intervention, the location to apply cupping was identified by marking on the bicep muscle belly at around one third from the shoulder to the elbow cubital fossa of the dominant arm. During the data collection, the participant was asked to maintain the posture of the forearm in supination, shoulder in abduction at 30 degrees and with the palm facing up. The pre-cupping and post-cupping of the muscle hemodynamic response was measured using NIRS for 5 and 10 minutes, respectively. The NIRS sensor band was placed on the biceps muscle. A bandage was used to wrap the sensor band to the arm (Fig 2).

### Data analysis

The pre-cupping measurement of muscle hemodynamic response was used as the baseline for determining the concentration change in oxyhemoglobin (Δ[HbO_2_]) and deoxy-hemoglobin (Δ[Hb]) after cupping therapy. In this study, the baseline measurement was 5 minutes. Blood volume is defined as a summation of oxyhemoglobin and deoxy-hemoglobin (Δ[HbO_2_] + Δ[Hb]). Oxygenation is defined as the difference between oxyhemoglobin and deoxy-hemoglobin (Δ[HbO_2_]—Δ[Hb]). The two-way analysis of variance (ANOVA) with repeated measures was used to examine the main effect of cup size and location factors and the interaction effect between the cup size and location factors. The test of distribution was performed to determine whether the data were normally distributed. For post-hoc comparisons, Bonferroni correction was used. The dependent variables were oxyhemoglobin (Δ[HbO_2_] in μM), deoxy-hemoglobin (Δ[Hb] in μM), blood volume (Δ[HbO_2_] + Δ[Hb] in μM), and oxygenation (Δ[HbO_2_]—Δ[Hb] in μM). The independent variables were the cup size (35, 40, and 45 mm) and location (the area inside the cup (NIRS channels 9, 10, 11, and 12 in this study) and the area outside the cup (NIRS channels 7, 8, 13, and 14 in this study) (Fig 1). The significance level was set at *P* <0.05. All NIRS analyses were performed using MATLAB (2022a, The MathWorks Inc., Natick, MA), and the statistical analyses were performed using SPSS (version 29, IBM Corp., Armonk, NY).

## Results

Eighteen healthy participants (12 females, 6 males) were enrolled in this study and their characteristics were (mean ± S.D.): age 25.0 ± 4.6 years, BMI 22.8 ± 3.5 kg/m^2^, arm circumference 27.1 ± 3.6 cm, arm length 32.6 ± 1.4 cm, and cupping location 10.7 ± 1.4 cm (Table 1). All participants were right handed. No participant reported adverse events after cupping therapy. All participants reported higher subjective pain feeling during cupping therapy using the 35-mm cup compared with the 40-mm and 45-mm cups.

**Table 1:** The characteristics of the participants.

	Age (years)	Height (cm)/ Weight (kg)	BMI (kg/m^2^)	Arm Circumference (cm)	Arm Length (cm)	Cupping Location ^a^ (cm)
Mean (S.D.)	24.63 (4.57)	165.18 (8.59) / 62.89 (14.33)	22.8 (3.52)	27.12 (3.62)	32.63 (1.37)	10.74 (1.37)

^a^ The distance between the cubital fossa of the elbow and the location of the cup.

The test of sphericity of SPSS indicated no violation of the assumption of normal distribution in oxyhemoglobin, deoxy-hemoglobin, blood volume and oxygenation. Therefore, parametric statistics was used in this study. The hemodynamic responses including oxyhemoglobin, deoxy-hemoglobin, blood volume and oxygenation after three protocols of cupping therapy are presented in Table 2. Data are expressed in means ± S.D (unit in μM).

**Table 2:** Hemodynamic response to three cupping sizes on the areas inside and outside the cup.

		Cup Size		
		35mm	40mm	45mm
**Inside the cup**	ΔOxyhemoglobin (μM)	3.58±2.81	2.03±1.75	6.52±4.30
	ΔDeoxy-hemoglobin (μM)	0.76±1,70	-0.27±1.93	2.16±2.05
	ΔBlood volume (μM)	4.34±3.62	1.75±3.03	8.69±5.67
	ΔOxygenation (μM)	2.82±2.91	2.30±2.10	4.36±3.64
**Outside the cup**	ΔOxyhemoglobin (μM)	2.68±1.76	2.16±1.38	4.95±2.73
	ΔDeoxy-hemoglobin (μM)	0.53±1.52	0.39±1.44	1.69±2.15
	ΔBlood volume (μM)	3.21±2.57	2.56±2.27	6.64±4.40
	ΔOxygenation (μM)	2.15±2.05	1.77±1.67	3.26±2.17

### Oxyhemoglobin

**Fig 3:**
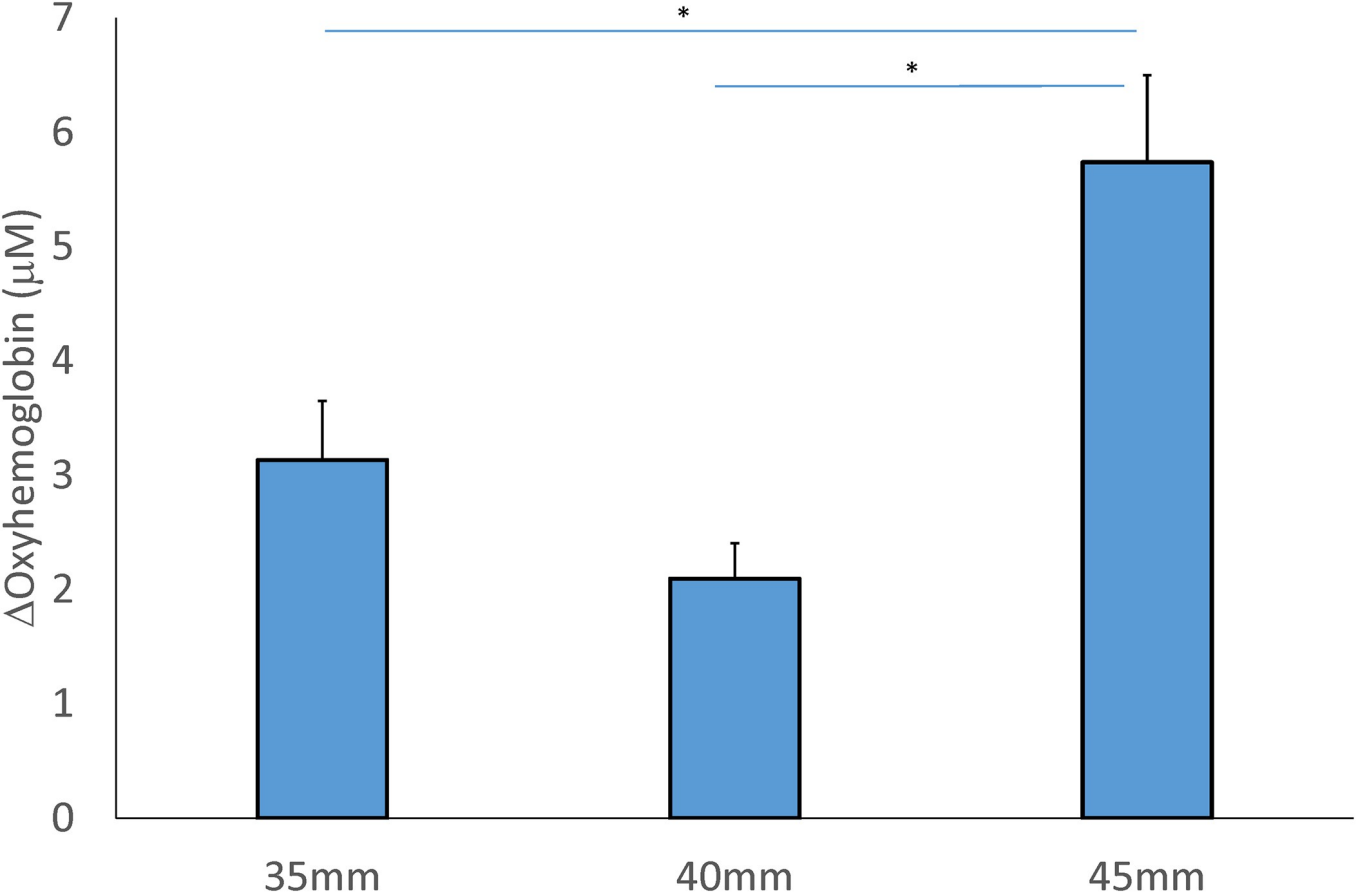
Comparison of the mean change of the concentration in oxyhemoglobin after 3 sizes of cupping cup. Values (post-cupping–pre-cupping oxyhemoglobin) are presented in means ± SE. (* *P* < 0.05).

The two-way repeated measures ANOVA shows no interaction between the cupping size and location factors (F = 2.467, *P* = 0.100), and a main effect of the cup size and location factors in oxyhemoglobin in Tables 2 and 3. For the cupping size factor, the 45-mm cup (5.738±0.760 μM) resulted in a significant increase in oxyhemoglobin compared to the 40-mm (2.095±0.312 μM, *P* < 0.001) and 35-mm (3.134±0.515 μM, *P* < 0.01) cup (Fig 3).

**Table 3:** Statistical results of the two-way ANOVA with repeated measures on the main effects of cup size and location and the interaction effect between cup size and location.

		F Values	*P* Values	Effect Size
**Cup Size × Location**	ΔOxyhemoglobin	2.467	0.100	0.127
	ΔDeoxy-hemoglobin	3.564	0.039*	0.173
	ΔBlood Volume	3.607	0.038*	0.175
	ΔOxygenation	0.410	0.667	0.024
**Cup Size**	ΔOxyhemoglobin	13.175	0.001*	0.437
	ΔDeoxy-hemoglobin	5.464	0.009*	0.243
	ΔBlood Volume	12.506	0.001*	0.424
	ΔOxygenation	3.739	0.034*	0.180
**Location**	ΔOxyhemoglobin	6.211	0.023*	0.268
	ΔDeoxy-hemoglobin	0.003	0.959	0.000
	ΔBlood Volume	2.800	0.113	0.141
	ΔOxygenation	8.296	0.010*	0.328

### Deoxy-hemoglobin

**Fig 4:**
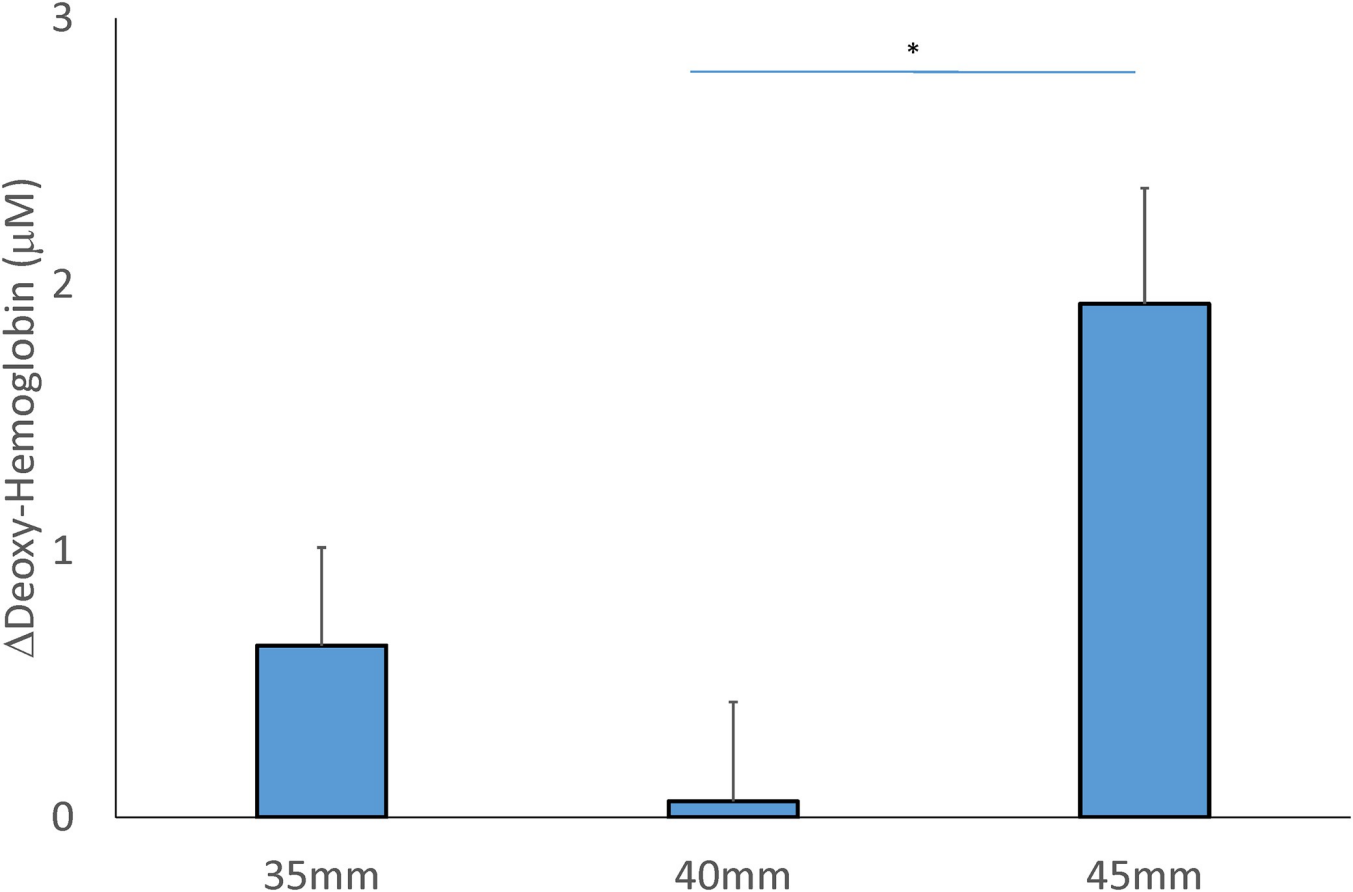
Comparison of the mean change of the concentration in deoxy-oxyhemoglobin after 3 sizes of cupping cup. Values (post-cupping–pre-cupping deoxy-hemoglobin) are presented in means ± SE. (* *P* < 0.05).

A significant interaction of the cup size and location factors was observed in deoxy-hemoglobin (F = 3.564, *P* = 0.039*, effect size = 0.173). A main effect of the cup size is found in deoxy-hemoglobin, but no effect of the location factor (Tables 2 and 3). For the cupping size factor, the 45-mm cup (1.928±0.434 μM) resulted in a significant increase in deoxy-oxyhemoglobin compared to the 40-mm cup (0.060±0.373 μM, *P* < 0.05) (Fig 4).

### Blood volume

**Fig 5:**
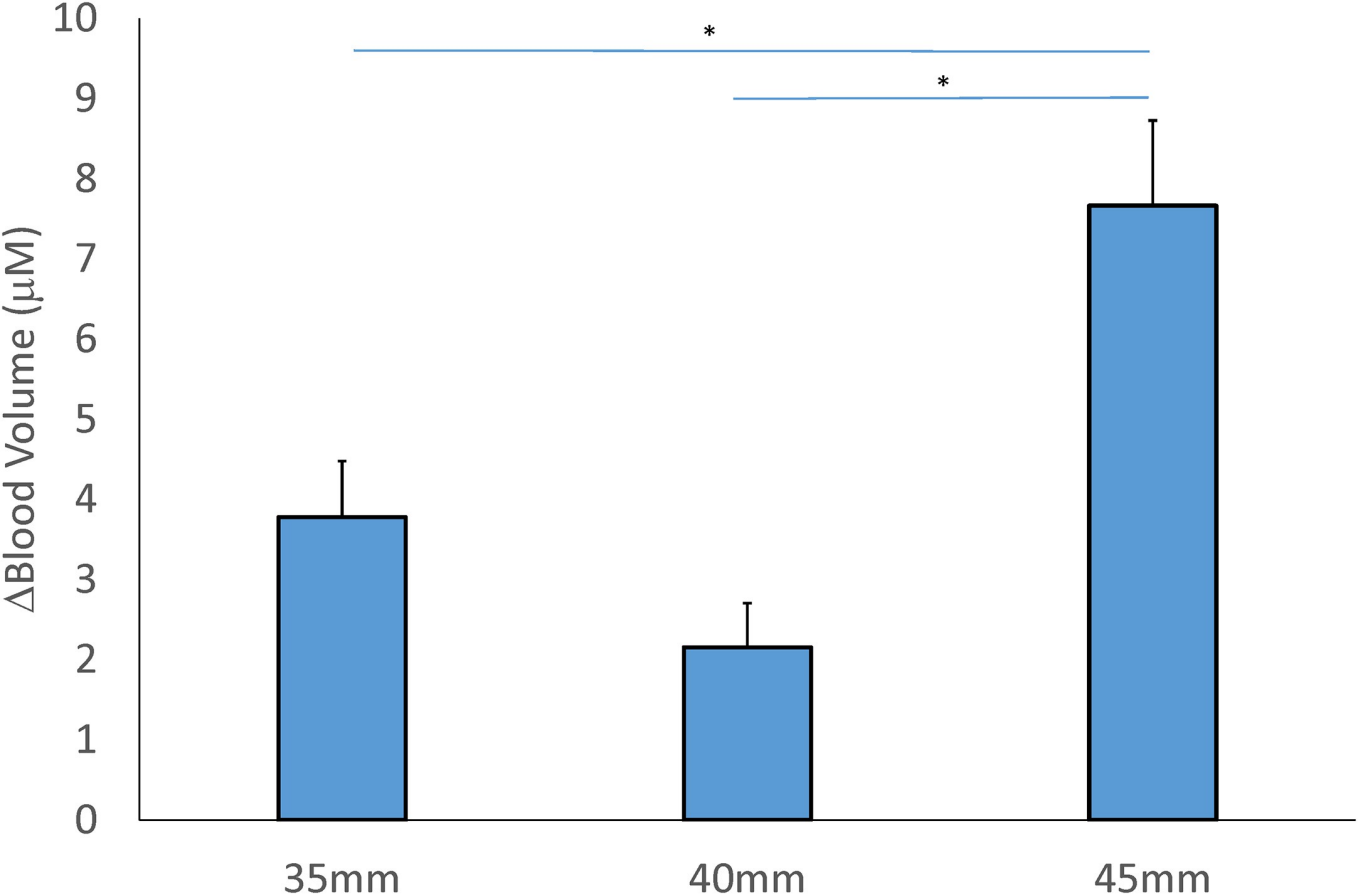
Comparison of the mean change of the concentration in blood volume after 3 sizes of cupping cup. Values (post-cupping–pre-cupping blood volume) are presented in means ± SE. (* *P* < 0.05).

A significant interaction of the cup size and location factors was observed in blood volume (F = 3.607, *P* = 0.038*, effect size = 0.175). There was a significant main effect of cup size factor on blood volume, but no effect of the location factor (Tables 2 and 3). For the cupping size factor, the 45-mm cup (7.666±1.061 μM) resulted in a significant increase in blood volume compared to the 40-mm (2.155±0.553 μM, *P* < 0.001) and 35-mm (3.779±0.698 μM, *P* < 0.01) cups (Fig 5).

### Oxygenation

**Fig 6:**
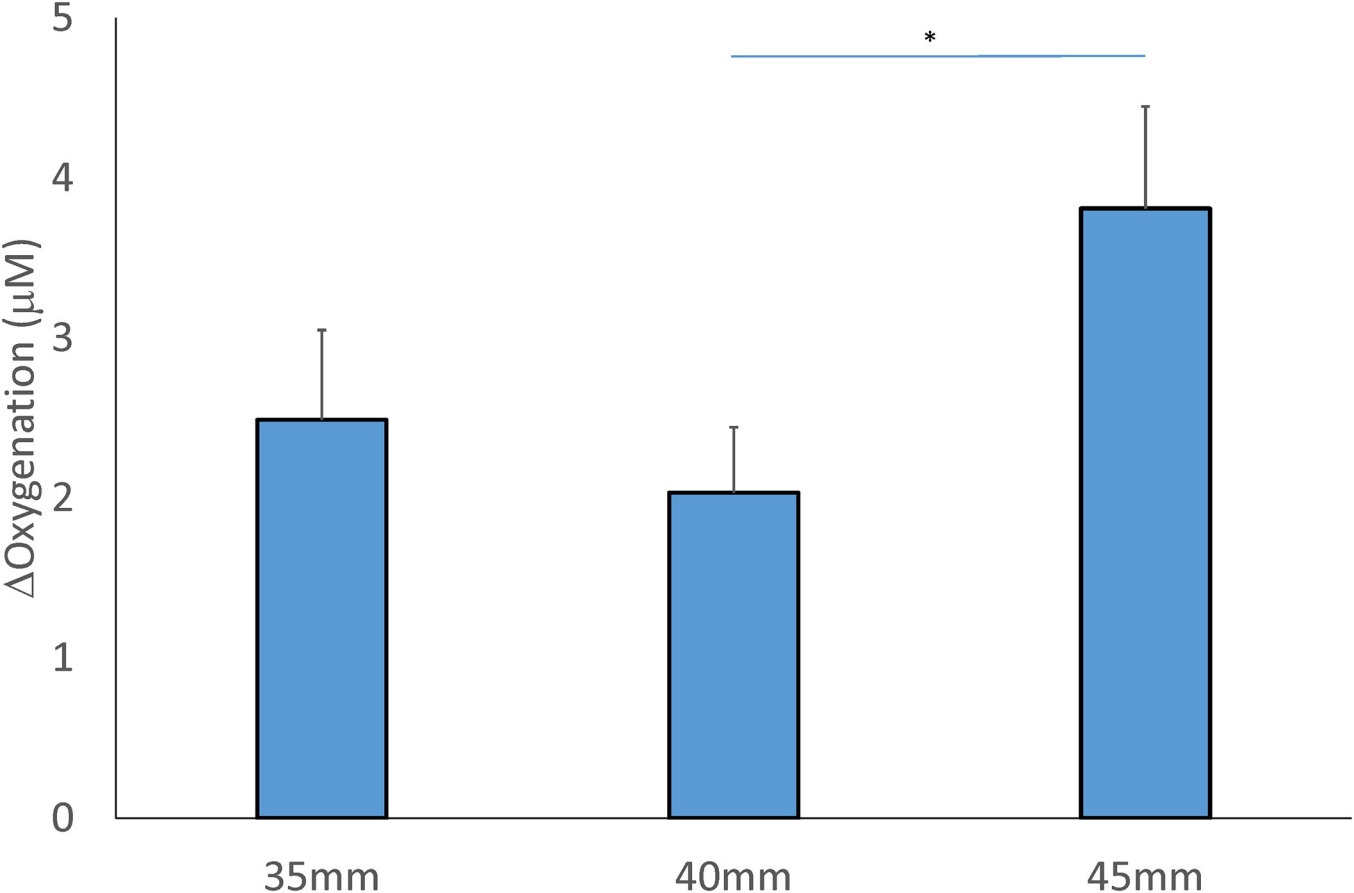
Comparison of the mean change of the concentration in oxygenation after 3 sizes of cupping cup. Values (post-cupping–pre-cupping oxygenation) are presented in means ± SE. (* *P* < 0.05).

There is no interaction between the cup size and location factors (F = 0.410, *P* = 0.667), and a main effect of the cup size and location factors was found in oxygenation (Tables 2 and 3). For the cupping size factor, the 45-mm cup (3.810±0.638 μM) resulted in a significant increase in oxygenation compared to the 40-mm cup (2.034±0.409 μM, *P* < 0.05) (Fig 6).

## Discussion

This study demonstrates that the cupping size could effectively affect muscle hemodynamic response, including oxyhemoglobin and deoxy-hemoglobin, in both the area inside and outside the cup. The results provide initial evidence showing the main effect of the cupping size on the muscle hemodynamic responses. Our results also demonstrated a significant interaction effect between the cupping size and location (areas inside and outside the cup) factors on muscle deoxy-hemoglobin and blood volume but no interaction effect between the cupping size and location factors on oxyhemoglobin and oxygenation.

Research studies have demonstrated that cupping therapy could effectively increase muscle blood volume [26]. However, it is unclear what intensity (in terms of cupping pressure, duration and size) of cupping therapy could effectively induce a hyperemic response for improving blood flow of the treated area. We speculated that an insufficient intensity of cupping therapy could not induce a hemodynamic response of the muscle. In this study, we demonstrated that the 45-mm cup could stimulate a larger increase in oxyhemoglobin compared to the 40-mm and 35-mm cups. This finding is consistent with our hypothesis because under the same negative pressure (-300 mmHg) and duration (5 min) of cupping therapy, a larger cup could stimulate a larger area of the muscle for inducing a larger hyperemic response. This is consistent with previous studies on assessing the cup size effect on the skin blood flow response using laser Doppler flowmetry [14] and the muscle stiffness using elastographic ultrasound [2].

Using the two-way factorial design with the main factors as the cupping size (35, 40, and 45 mm) and the location (areas inside and outside the cup), our results indicate that the cupping size is a significant main factor on oxyhemoglobin, deoxy-hemoglobin, blood volume and oxygenation. This is a significant finding because an appropriate cupping size can induce the hemodynamic response in both the areas inside and outside the cup. This demonstrates the benefits of cupping therapy on improving the hemodynamic response is not limited to the area inside the cup. In this study, we compared the effect of three common sizes of cupping therapy on muscle hemodynamic responses. The results demonstrated that all three cup sizes (35, 40, and 45 mm) can significantly improve oxyhemoglobin and blood volume compared to pre-cupping. (The results presented in this study are the relative change after cupping therapy compared to pre-cupping hemodynamic value.) The 45-mm cup is more effective on improving hemoglobin compared to the 35-mm and 40-mm cups.

In our previous study [14], the 35-mm was least effective among the 3 cupping sizes on improving skin blood flow. In this study, the least effective size on modulating muscle blood responses was the 40-mm cup. All participants commented that the 35-mm cup was uncomfortable. We speculate that a small cup may cause more deformation of the sucked soft tissue and may induce a relatively intensive response to cupping therapy compared to a middle size cup such as the 40-mm cup in this study. This observation of a small cup size (35 mm in this study) and a large cup size (45 mm in this study) is more effective than the middle size (40 mm in this study) provides a new finding about the cup size effect. However, these findings do not conclude that the 40-mm cup is less effective compared with other cup sizes. Nevertheless, it remains largely unknown whether pain caused by a smaller cup and large deformation caused by a larger cup are mediating factors of muscle hemodynamic responses after cupping therapy. Further research studies are needed to examine whether pain perception would result in a larger hemodynamic response.

The interaction between the cupping size and the location is examined in this study. This is the first study to investigate the interaction effect of cup size and locations. Considering the impact between the cup size and the location, the interaction existed in deoxy-hemoglobin and total blood volume but not oxyhemoglobin and the oxygenation. Some research studies suggested that the change in deoxy-hemoglobin concentration is a better index to estimate the metabolic situation rather than the oxyhemoglobin or total blood volume in the tissue [23]. To date, there is no sufficient evidence to prove that the muscle metabolic system is improved after cupping therapy. From our previous studies [7,14], the metabolic control was demonstrated to modulate an increase in skin blood flow. The metabolic control is mainly mediated by the release of nitric oxide, a vasodilator. Cupping may cause the release of nitric oxide from the area inside the cup and after blood circulation is restored, these nitric oxide may bed transported to adjacent microvascular systems for causing a weaker vasodilatory response compared to the area inside the cup [27].

This study also demonstrated that oxyhemoglobin and the oxygenation concentrations inside the cupping cup are significantly higher than those immediately outside the cupping cup (the equal areas were assessed using 4 channels of NIRS). The oxygenation is an index produced from the sum of oxyhemoglobin and deoxy-hemoglobin. This result implies that the oxyhemoglobin increases more than deoxy-hemoglobin at the area inside the cup compared with the area outside the cup. On the other hand, the changes in the concentration of deoxy-hemoglobin between the inside and outside the cup are similar. It may be caused by the increased metabolism rate.

Researchers suggested that deoxy-hemoglobin could be an index to measure the change of tissue activation [28]. Also, oxygenation is described as the ability to send oxygen to the tissue [17]. Both deoxy-hemoglobin and oxygenation might be better parameters to demonstrate the performance of the metabolism system. In our research, the shift in deoxy-hemoglobin shows that the larger cup size can induce a more robust response. On the other hand, a 45-mm cup should be a better choice to promote the metabolism system of biceps muscle tissue. Although the 40 mm was less effective on inducing a hyperemic response compared to the 35-mm and 45-mm cup, the trend supports our hypothesis. It is needed to further investigate the mechanisms of action associated with a larger hemodynamic response from the 45-mm and 35-mm cup compared with the 40-mm cup. The authors speculate that an appropriate ratio of the cup size to the treated area may exist and determine the hemodynamic response. Furthermore, increasing metabolism could improve muscle recovery, but clearing the waste might take one day [1]. Oxygenation represents the balance between oxygen delivery and uptake. The increased value of oxyhemoglobin and deoxy-hemoglobin of the biceps muscle in response to cupping therapy indicated that cupping therapy can improve the oxygen delivery and oxygen utilization of the muscle. Our findings further demonstrate that both cup size and location factors affect muscle blood volume and oxygenation. Cupping therapy has capacity to become a rehabilitation intervention to benefit result for improving muscle healing.

Our findings have clinical implications. Because our result indicate that a larger cup could induce a larger hemodynamic response, it is important for a clinician to choose a larger cup when the target area is not well defined, such as low back pain. However, the selection of a larger cup should be cautious when treating the neck and forearm, a smaller cup may be more appropriate. The selection of cup size remains an important topic for research. Our results also indicate that even on the area outside the cup rim, cupping therapy could still stimulate an increase in the muscle hemodynamic response. This indicates that cupping therapy not only benefit the area inside the cup, but also the area outside the cup. Although it requires further investigation on whether an increased blood flow could lead to better tissue healing in long term, an increase in blood flow and oxygenation is usually considered as an effective intervention. For example, negative pressure is used on wound healing by increasing blood flow around the wound [29,30].

There are limitations in this study. First, we did not have a control group for receiving sham cupping therapy. Because we blinded the selection of cup size to the participant and used a repeated measures design for testing 3 cup sizes, we expect that the difference measured from NIRS would be attributed to cupping therapy. This study design would minimize the influence from other factors. Second, all three protocols were conducted in the same day. Although a wash-out period of 40 minutes and counter-balanced design of the testing order of 3 protocols were implemented, the carry-over and time effect from cupping therapy may exist. Insufficient recovery from the previous cupping therapy may reduce the degree of hyperemic response. Also, the interaction between time and treatment factors could affect the outcome. Further research is needed to examine these factors of cupping therapy. Third, we did not exclude the female physiological period. This may confound the outcome. Last, we investigated the acute effect of cupping therapy on the muscle hemodynamic response. It is unclear whether these observed response would persist over a longer period of time. Future research may need to investigate the long-term effect of cupping therapy on the muscle hemodynamic response.

## Conclusion

This study presents the main effect of cupping size and location of cupping therapy and their interaction on the muscle hemodynamic response using a multi-channel NIRS system. Our results provide a quantitative evidence of a larger cup (the 45-mm circular cup in inner diameter) resulting in a larger hemodynamic responses (oxyhemoglobin and deoxy-hemoglobin) of the biceps muscle compared to smaller sizes (the 35-mm and 40-mm cup). Our study indicates that the muscle hemodynamic response to cupping therapy is determined by the combination of cupping size and location. The use of NIRS technology could help establish the dose-response relationship between the cupping size and hemodynamic response of the muscle.

## Supporting information

S1 File (PDF)
