# Supplementary material for: Application of near-infrared spectroscopy to assess the effect of the cupping size on the spatial hemodynamic response from the area inside and outside the cup of the biceps
Source: PLoS One. 2024 May 9;19(5):e0302828. doi: 10.1371/journal.pone.0302828 (PMC11081366; doi:10.1371/journal.pone.0302828)
Supplement: S1 File — (PDF) [file pone.0302828.s001.pdf]

NIRS SPSS Size.sav

	Oxy_35	Oxy_40	Oxy_45	Deoxy_35	Deoxy_40	Deoxy_45	Volume_35	Volume_40
1	2.30	1.98	2.27	2.30	-.22	-.88	4.59	1.76
2	8.51	1.04	7.64	-.77	-4.15	-.77	7.74	-3.11
3	-.40	1.33	6.85	-.75	-.69	3.80	-1.15	.64
4	.16	3.82	2.74	-1.57	1.06	1.51	-1.41	4.87
5	4.35	5.13	16.31	2.31	.89	3.88	6.66	6.02
6	4.88	.18	16.22	1.55	-2.43	3.67	6.42	-2.25
7	2.03	3.17	2.79	.40	1.89	1.28	2.42	5.06
8	4.90	-1.09	3.74	-.15	1.18	-.71	4.74	.09
9	1.29	4.78	2.19	.24	1.40	.10	1.54	6.18
10	1.81	3.31	5.20	.23	.92	1.79	2.04	4.23
11	1.27	2.39	6.17	-.78	-.32	6.92	.49	2.07
12	4.65	-.28	7.29	2.83	.43	2.23	7.47	.15
13	6.03	-.40	4.94	5.29	-3.84	2.36	11.32	-4.24
14	5.76	2.29	5.60	-.50	1.41	3.22	5.25	3.70
15	2.21	3.46	6.07	-.45	-3.70	2.73	1.76	-.25
16	2.21	1.92	8.67	2.17	.56	3.22	4.38	2.48
17	10.17	2.26	10.82	.44	.45	4.12	10.61	2.71
18	2.38	1.21	1.93	.93	.26	.44	3.32	1.47

...	Volume_45	Oxygen_35	Oxygen_40	Oxygen_45	O_Oxy_35	O_Oxy_40	O_Oxy_45	O_Deoxy_35
1	1.39	.00	2.20	3.14	1.97	1.34	3.25	1.35
2	6.87	9.28	5.19	8.42	4.88	3.48	9.76	-.46
3	10.66	.35	2.03	3.05	1.23	3.46	6.63	1.01
4	4.25	1.73	2.76	1.23	.38	3.49	.16	-1.43
5	20.19	2.05	4.23	12.43	2.31	2.91	10.05	2.07
6	19.89	3.33	2.62	12.55	2.65	2.63	7.53	.05
7	4.07	1.63	1.28	1.50	2.45	1.75	3.74	.41
8	3.03	5.05	-2.27	4.45	4.00	1.73	2.35	-1.51
9	2.29	1.05	3.37	2.08	2.07	4.94	3.89	1.33
10	6.99	1.59	2.39	3.41	.90	1.47	3.29	.11
11	13.10	2.05	2.71	-.75	.55	-.12	4.46	-1.40
12	9.52	1.82	-.71	5.05	4.34	1.16	5.29	2.49
13	7.31	.74	3.44	2.58	4.87	-.33	3.55	4.39
14	8.81	6.26	.88	2.38	1.96	2.18	8.54	-.52
15	8.80	2.66	7.16	3.34	4.35	1.61	6.21	-.71
16	11.90	.05	1.36	5.45	1.20	2.02	5.36	1.07
17	14.94	9.74	1.80	6.69	6.63	4.03	3.43	.24
18	2.37	1.45	.95	1.48	1.59	1.16	1.66	1.03

NIRS SPSS Size.sav

	O_Deoxy_40	O_Deoxy_45	O_Volume_35	O_Volume_40	O_Volume_45	O_Oxygen_35	O_Oxygen_40	O_Oxygen_45
1	-.50	.81	3.32	.84	4.06	.61	1.83	2.44
2	-1.79	4.20	4.42	1.68	13.95	5.34	5.27	5.56
3	1.53	4.58	2.24	4.99	11.20	.22	1.93	2.05
4	1.34	-2.33	-1.06	4.84	-2.17	1.81	2.15	2.48
5	-.07	4.17	4.37	2.84	14.22	.24	2.98	5.88
6	-1.06	-.52	2.70	1.57	7.01	2.59	3.69	8.05
7	1.44	1.92	2.86	3.19	5.66	2.05	.31	1.81
8	1.86	-1.22	2.49	3.60	1.13	5.52	-.13	3.56
9	.96	.19	3.40	5.90	4.08	.73	3.99	3.70
10	-.68	.03	1.01	.78	3.32	.79	2.15	3.27
11	.68	5.48	-.85	.55	9.94	1.95	-.80	-1.02
12	2.15	1.27	6.83	3.31	6.57	1.85	-1.00	4.02
13	-2.22	1.26	9.26	-2.55	4.81	.47	1.89	2.29
14	2.37	3.64	1.44	4.55	12.17	2.47	-.19	4.90
15	-1.16	1.17	3.64	.45	7.38	5.06	2.77	5.04
16	.55	2.02	2.28	2.57	7.38	.13	1.47	3.34
17	2.19	3.18	6.88	6.22	6.61	6.39	1.85	.24
18	-.50	.62	2.62	.66	2.27	.56	1.66	1.04
